# The isotropic fractionator provides evidence for differential loss of hippocampal neurons in two mouse models of Alzheimer's disease

**DOI:** 10.1186/1750-1326-7-58

**Published:** 2012-11-22

**Authors:** Hannah Brautigam, John W Steele, David Westaway, Paul E Fraser, Peter H St George-Hyslop, Sam Gandy, Patrick R Hof, Dara L Dickstein

**Affiliations:** 1 Fishberg Department of Neuroscience, Icahn School of Medicine at Mount Sinai, New York, NY, 10029, USA; 2 Friedman Brain Institute, Icahn School of Medicine at Mount Sinai, New York, NY, 10029, USA; 3 Department of Neurology, Icahn School of Medicine at Mount Sinai, New York, NY, 10029, USA; 4 Department of Psychiatry, Icahn School of Medicine at Mount Sinai, New York, NY, 10029, USA; 5 Alzheimer’s Disease Research Center, Icahn School of Medicine at Mount Sinai, New York, NY, 10029, USA; 6 James J. Peters VA Medical Center, Bronx, NY, 10468, USA; 7 Laboratory of Molecular and Cellular Neuroscience, Rockefeller University, New York, NY, 10065, USA; 8 Centre for Prions and Protein Folding Diseases, University of Alberta, Edmonton, AB, T6G 2M8, Canada; 9 Tanz Centre for Research in Neurodegenerative Diseases, Departments of Medical Biophysics and Medicine (Neurology), University of Toronto, Toronto, ON, M5S 3H2, Canada; 10 Cambridge Institute for Medical Research, University of Cambridge, Cambridge, CB2 0XY, UK; 11 Department of Neuroscience, Leon and Norma Hess Center for Science and Medicine, 10th Floor, 1470 Madison Avenue, New York, NY, 10029, USA

**Keywords:** Alzheimer’s disease, Mouse models, Amyloid beta (Aβ), Isotropic fractionator, Neuronal loss

## Abstract

**Background:**

The accumulation of amyloid beta (Aβ) oligomers or fibrils is thought to be one of the main causes of synaptic and neuron loss, believed to underlie cognitive dysfunction in Alzheimer’s disease (AD). Neuron loss has rarely been documented in amyloid precursor protein (APP) transgenic mouse models. We investigated whether two APP mouse models characterized by different folding states of amyloid showed different neuronal densities using an accurate method of cell counting.

**Findings:**

We examined total cell and neuronal populations in Swedish/Indiana APP mutant mice (TgCRND8) with severe Aβ pathology that includes fibrils, plaques, and oligomers, and Dutch APP mutant mice with only Aβ oligomer pathology. Using the isotropic fractionator, we found no differences from control mice in regional total cell populations in either TgCRND8 or Dutch mice. However, there were 31.8% fewer hippocampal neurons in TgCRND8 compared to controls, while no such changes were observed in Dutch mice.

**Conclusions:**

We show that the isotropic fractionator is a convenient method for estimating neuronal content in milligram quantities of brain tissue and represents a useful tool to assess cell loss efficiently in transgenic models with different types of neuropathology. Our data support the hypothesis that TgCRND8 mice with a spectrum of Aβ plaque, fibril, and oligomer pathology exhibit neuronal loss whereas Dutch mice with only oligomers, showed no evidence for neuronal loss. This suggests that the combination of plaques, fibrils, and oligomers causes more damage to mouse hippocampal neurons than Aβ oligomers alone.

## Findings

**Figure 1:**
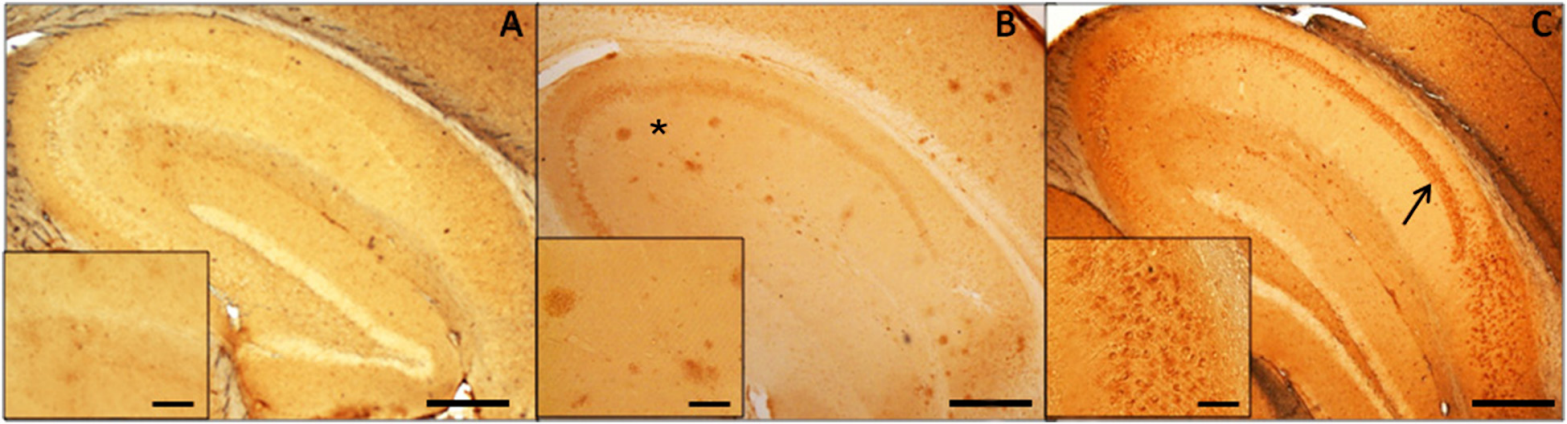
**APP/Aβ pathology in the hippocampus of TgCRND8 mutant APP and Dutch mutant APP transgenic mice.** Aβ and APP species were visualized using the 6E10 antibody. Extracellular amyloid plaques (asterisk) are evident in (**B**) TgCRND8 APP transgenic mice (3.5 months old), while (**C**) Dutch mutant APP (17 months old) exhibit intracellular APP/Aβ-like immunoreactivity (APP/Aβ-LIR; arrow). No APP/Aβ-LIR is seen when 6E10 is used to immunostain brain of a wild type mouse (**A**). Inset panels represent higher magnification of 6E10 immunoreactivity of plaques in TgCRND8 mutant APP mice and intracellular APP/Aβ-LIR in Dutch mutant APP mice. Scale bars represent 500 μm. Scale bars in insets represent 100 μm.

Alzheimer’s disease (AD) is a progressive neurodegenerative disorder characterized by the presence of extracellular amyloid beta (Aβ) plaques, neurofibrillary tangles, and severe loss of synapses and neurons 1. One of the limitations of AD mouse models has been the absence of cell loss in many of them 2,3,4, although systematic head-to-head comparison of neuronal loss in mouse models has been hindered by the fact that high-quality morphometry is not routinely available or achieved. Furthermore, there are a number of non-stereologic approaches, prone to observer biases, which are often employed but fail to produce comparable results, within a laboratory as well as across studies. As a result, reliable quantification of neuronal loss has not been routinely included in the characterization of mouse models. Absence of data on neuronal loss reduces the usefulness in specifying how particular transgenes and interventions impact neuronal integrity. Here, we examined changes in total cell (i.e., neurons and glia) and total neuronal numbers and densities in two mouse models of AD harboring different APP mutations and exhibiting different panoplies of Aβ biophysical phenotypes: the TgCRND8 mouse and the Dutch APP mouse. TgCRND8 mice express the Swedish (K670M/N671L) and Indiana (V717F) APP mutations, and exhibit increases in total production of Aβ and in the Aβ_42_/Aβ_40_ ratio, leading to accumulation of intraneuronal Aβ, Aβ amyloid plaques, soluble oligomeric Aβ (oAβ), and insoluble fibrillar Aβ (fAβ) 5. The Dutch mouse expresses the E693Q mutation that favors the production of oAβ over fAβ and accumulates intraneuronal Aβ and oAβ 3(Additional file 1: Table S1; Figure 1).

**Figure 2:**
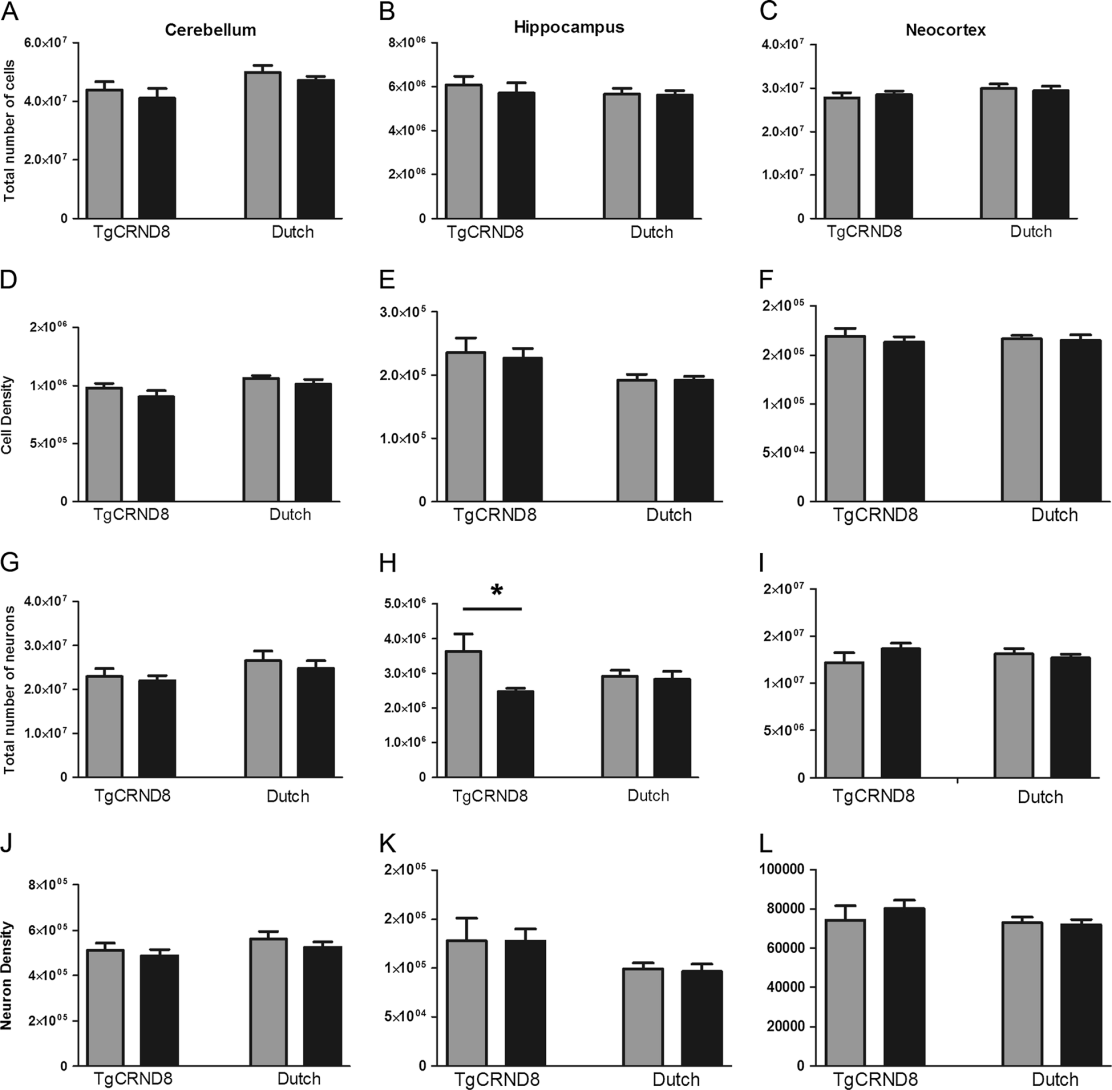
**TgCRND8 mutant APP transgenic mice exhibit significantly lower neuronal numbers and densities as compared to Dutch mutant APP transgenic mice.** There was a significant difference observed in neuronal numbers in the hippocampus between TgCRND8 mutant APP mice and their control littermates (**H**). Left column represents TgCRND8 mutant APP mice (black) and their littermate controls (grey) and the right column represents the Dutch mutant APP mice (black) and their littermate controls (grey). Note that the scale of the y-axis changes among the graphs. Values represent mean ± SEM. *p < 0.05.

We employed the isotropic fractionator method 6, and quantified cell and neuronal populations in these two different APP mouse lines that we predicted would demonstrate different levels of neuronal integrity. When comparing our total cell numbers to published estimates from wildtype mouse brains, we found that they were comparable to those reported for whole brain using the isotropic fractionator 7, and for specific brain regions such as the cerebellum, as estimated by isotropic fractionator and flow cytometry 8, and the hippocampus as assessed by traditional stereology 9,10,11,12(Table 1). When assessing total cell and neuron populations, we found no differences in total and regional brain weights between transgenic mice and their respective littermates. We also did not find any differences in total cell counts or densities between TgCRND8 mutant APP mice and controls (Figure 2A-F). However, we observed significantly fewer (31.8%) hippocampal neurons between TgCRND8 mutant APP mice and their non-transgenic littermates (*t*_(12)_ = 2.391, p = 0.033; Figure 2H). No significant differences were observed in total neuronal counts or densities in neocortex or cerebellum (Figure 2G, I-L). When we studied Dutch mutant APP mice, we found no significant differences from controls in total cell count, neuronal cell count, cell density, or neuronal density (Figure 2). We did not observe neuronal density changes in the neocortex, perhaps because of region- or even layer-specific vulnerability of select neuronal populations. We did not assess neuron numbers in specific neocortical regions and, as such, any locally restricted loss may have been undetected. We also analyzed mice at only one time point and it is possible that neocortical neuronal loss may appear at later stages.

**Table 1:** Summary of cell and neuron counts from different studies according to brain region and counting method

**Method**	**Genetic background**	**Brain region**	**Cell or neuron number**	**Age of animals and transgene(s) harbored**	**Reference**
Isotropic Fractionator	TgCRND8 (C3H/He-C57BL/6)	Whole brain	100.10 ± 7.90 x 10^6^	6 months; K670M/N671L/V717F APP	Present study
	Dutch (C57BL/6J)				
Isotropic Fractionator	Swiss Webster	Whole brain	108.90 ± 16.25 x 10^6^	2-5 months	7
Isotropic Fractionator	TgCRND8 (C3H/He-C57BL/6)	Cerebellum	43.92 ± 2.78 x 10^6^	6 months; K670M/N671L/V717F APP	Present study
	Dutch (C57BL/6J)				
Isotropic Fractionator	Swiss Webster	Cerebellum	49.17 ± 5.32 x 10^6^	2-5 months	7
Isotropic Fractionator/Flow Cytometry	C57Bl/6J	Cerebellum	44.03 ± 0.42 x 10^6^	60-100 days	8
Isotropic Fractionator	TgCRND8 (C3H/He-C57BL/6)	Hippocampus	31.8% decrease in neurons	6 months; K670M/N671L/V717F APP	Present Study
Isotropic Fractionator	Dutch (C57BL/6J)	Hippocampus	No decrease in neurons	18 month Dutch E693Q APP mice	Present study
Stereology	C57BL/6	CA1	~25% decrease in neurons	14-18 month-old TgAPP23 (K670M/N671L) mice	9
Stereology	APP/ PS1^M146L^ (CBA - 12.5% × C57Bl6 - 87.5%)	CA1-CA3	~30% decrease in neurons	17 month APP (751^*SL*^) and presenilin 1 (PS1^M146L^) mice	10
	Controls (C57BL/6)				
Stereology	(C57BL/6 50%-CBA 25%–129SV 25%)	CA1/2	~50% decrease in neurons	10 month APP^SL^PS1KI (APP K670N/M671L, and V717I; PS1 M233T/L235P knock-in)	11
Stereology	C57BL/6J	CA1	33% decrease in neurons	6 month APP/PS1KI	12
				(APP K670N/M671L, and V717I; PS1 M233T/L235P knock-in)	

Aberrant accumulation of brain Aβ over decades has been proposed to result in synaptic and neuronal loss associated with the progression of AD (for review see 13). Evidence indicates that accumulation of soluble oAβ relates to cognitive impairment in AD patients 14and AD mouse models 2,3,15. However, the possibility that the severity of neurotoxicity and neuronal loss may be related to levels of Aβ solubility or confirmation (oAβ or fAβ) remains unclear. Using high-resolution 3D reconstructions, intraneuronal accumulation of fAβ has been observed by some investigators who proposed that this fAβ leads to the disruption and degeneration of spines and neurites, and ultimately to neuronal death 16, even though other investigators have reported that oAβ alone is sufficient for behavioral dysfunction 15.

Importantly, other studies have demonstrated reduced neuron densities in various lines of APP mice following onset of plaque pathology. These observations were primarily derived from the hippocampus and are similar in magnitude to the loss we report in TgCRND8 mice, albeit using different counting methods (Table 1). Using a stereologic approach, Calhoun et al. (1998) found a ~25% loss of CA1 neurons in TgAPP23 mice, while Schmitz et al. (2004) found a ~30% loss of neurons in the CA1-3 regions in an APP/PS1 double mutant mouse, and Breyhan et al. (2009) found a 33% loss of CA1 pyramidal neurons in APP/PS1 knock-in mice. Our results indicate that, in wildtype mice, estimates derived from the isotropic fractionator are generally comparable to stereologic estimates. Most importantly, we observed an apparent neuronal loss in TgCRND8 mice that form oAβ, insoluble fAβ, and amyloid plaques, but not in Dutch mice that produce only oAβ. It is evident that both oAβ and fAβ are toxic and implicated in the neurodegeneration process. However, various Aβ species may impair neuronal function via different mechanisms, and clarification of this point merits further investigation.

## Methods

### Animals

Eight 6-month-old TgCRND8 APP^K670N/M671L/V717F^ mice and seven wildtype littermates, as well as nine 18-month-old Dutch APP^E639Q^ mice and eight of their littermates were used in this study. These ages were chosen as previous studies demonstrated clear behavioral impairments and amyloid deposition in the TgCRND8 mice and intracellular accumulation in the Dutch mice at these time points 3,5. All mice used in the current study were group-housed, given *ad libitum* access to food and water, and housed under a 12-hour light/dark cycle. All animal procedures were conducted in accordance with the National Institute of Health Guidelines for the Care and Use of Experimental Animals and were approved by the Institutional Animal Care and Use Committee of the Icahn School of Medicine at Mount Sinai.

Animals were perfused as previously described 17. Brains were removed from the skull and postfixed in 4% PFA at 4°C. Brains were weighed, cut into half, and further dissected into three regions, cerebellum, hippocampus, and neocortex and weighed. Results were multiplied by 2 to obtain estimates of cell numbers for the entire brain. In addition, brains were immunostained with the anti-amyloid monoclonal antibody 6E10 (Covance) as described in 15.

### Isotropic fractionator

The isotropic fractionator is a fast and reliable technique for determining total cell and neuronal counts and cell densities in the brain. The procedure involves processing of highly anisotropic brain structures into homogeneous isotropic suspensions of cell nuclei that can be easily quantified 6. Because the estimates of cell counts are obtained separately from the determination of brain volume, the two measurements can be used in comparative studies of brain-volume variation (i.e., comparing across species or between different mouse lines 6). Briefly, postfixed samples were mechanically dissociated and homogenized in a solution of 40 mM sodium citrate and 1% Triton X-100 as previously described 6. The homogenates were collected and the homogenizer washed at least twice to collect any residual cells. To visualize nuclei and obtain total cell counts, 1 μl of 4’,6-diamidino-2-phenylindole (Sigma) was added to the cell suspension (volumes according to brain region; 10 ml for cerebellum and neocortex, 3 ml for hippocampus. The volume for each brain region was optimized so that at least 500 nuclei could be counted) To determine total neuron counts, 1 ml of each cell suspension was removed, washed with PBS, and centrifuged for 10 minutes at 4000 x g. Cells were then incubated in anti-NeuN antibody (1:200; Millipore) for 2 hours followed by incubation in a secondary anti-mouse IgG-Alexa-Fluor 594 for 1.5 hours (1:200; Millipore). Cells were then counted with a hemocytometer using a 20X/0.45 N.A., Plan-Apochromat objective on a Zeiss Axiophot microscope equipped with a motorized stage. All four quadrants in both the upper and lower grids of the hemocytometer were counted and averaged. Total cell numbers and neuronal numbers were calculated by multiplying the number of cells/ml by final volume. Densities were calculated by dividing the number of cells/ml by the mass of brain region.

### Statistics

All statistical analyses were performed using SPSS v19.0. Independent samples *t*-tests were used for comparison of TgCRND8 mutant APP or Dutch mutant APP mice to their respective littermates. Levene's test for homogeneity of variance was used for inclusion in parametric tests (*p* > 0.05). Significance for *t*-tests are reported with a *p* value < 0.05 using two-tailed tests with an α level of 0.05. Values represent mean ± SEM.

## Abbreviations

AD: Alzheimer’s disease; Aβ: Amyloid beta; APP: Amyloid precursor protein; PFA: Paraformaldehyde; PBS: Phosphate-buffered saline.

## Competing interests

The authors have no competing interests.

## Author’s contributions

HB, JWS, PRH, and DLD all contributed to the writing of the manuscript. HB and DLD performed the isotropic fractionator and analyses. SG (with Dr ME Ehrlich, Icahn School of Medicine at Mount Sinai) created the Dutch APP mouse model and DW, PEF, and PHH created the TgCRND8 APP mouse model. SG and PRH supervised the project and edited the manuscript. All authors have read and approved the manuscript.

## Supplementary Material

Additional file 1 **Table S1.** Comparison between the DutchAPP^E693Q^ mouse model and the TgCRND8APP^K670N/M671L/V7F^ mouse model 2,3,4,5. Click here for file

## References

[B1] Selkoe DJ Alzheimer's disease results from the cerebral accumulation and cytotoxicity of amyloid beta-protein J Alzheimers Dis 2001 3 75 80 12214075 10.3233/jad-2001-3111

[B2] Tomiyama T Matsuyama S Iso H Umeda T Takuma H Ohnishi K Ishibashi K Teraoka R Sakama N Yamashita T Nishitsuji K Ito K Shimada H Lambert MP Klein WL Mori H A mouse model of amyloid beta oligomers: their contribution to synaptic alteration, abnormal tau phosphorylation, glial activation, and neuronal loss in vivo J Neurosci 2010 30 4845 56 10.1523/JNEUROSCI.5825-09.2010 20371804 PMC6632783

[B3] Gandy S Simon AJ Steele JW Lublin AL Lah JJ Walker LC Levey AI Krafft GA Levy E Checler F Glabe C Bilker WB Abel T Schmeidler J Ehrlich ME Days to criterion as an indicator of toxicity associated with human Alzheimer amyloid-beta oligomers Ann Neurol 2010 68 220 30 20641005 10.1002/ana.22052 PMC3094694

[B4] Malthankar-Phatak GH Lin YG Giovannone N Siman R Amyloid deposition and advanced age fails to induce Alzheimer's type progression in a double knock-in mouse model Aging Dis 2012 3 141 55 22724075 PMC3377826

[B5] Chishti MA Yang DS Janus C Phinney AL Horne P Pearson J Strome R Zuker N Loukides J French J Turner S Lozza G Grilli M Kunicki S Morissette C Paquette J Gervais F Bergeron C Fraser PE Carlson GA George-Hyslop PS Westaway D Early-onset amyloid deposition and cognitive deficits in transgenic mice expressing a double mutant form of amyloid precursor protein 695 J Biol Chem 2001 276 21562 70 10.1074/jbc.M100710200 11279122

[B6] Herculano-Houzel S Lent R Isotropic fractionator: a simple, rapid method for the quantification of total cell and neuron numbers in the brain J Neurosci 2005 25 2518 21 10.1523/JNEUROSCI.4526-04.2005 15758160 PMC6725175

[B7] Herculano-Houzel S Mota B Lent R Cellular scaling rules for rodent brains Proc Natl Acad Sci U S A 2006 103 12138 43 10.1073/pnas.0604911103 16880386 PMC1567708

[B8] Surchev L Nazwar TA Weisheit G Schilling K Developmental increase of total cell numbers in the murine cerebellum Cerebellum 2007 1 6 10.1080/14734220601169699 17853078

[B9] Calhoun ME Wiederhold KH Abramowski D Phinney AL Probst A Sturchler-Pierrat C Staufenbiel M Sommer B Jucker M Neuron loss in APP transgenic mice Nature 1998 395 755 6 10.1038/27351 9796810

[B10] Schmitz C Rutten BP Pielen A Schafer S Wirths O Tremp G Czech C Blanchard V Multhaup G Rezaie P Korr H Steinbusch HW Pradier L Bayer TA Hippocampal neuron loss exceeds amyloid plaque load in a transgenic mouse model of Alzheimer's disease Am J Pathol 2004 164 1495 502 10.1016/S0002-9440(10)63235-X 15039236 PMC1615337

[B11] Casas C Sergeant N Itier JM Blanchard V Wirths O van der Kolk N Vingtdeux V van de Steeg E Ret G Canton T Drobecq H Clark A Bonici B Delacourte A Benavides J Schmitz C Tremp G Bayer TA Benoit P Pradier L Massive CA1/2 neuronal loss with intraneuronal and N-terminal truncated Abeta42 accumulation in a novel Alzheimer transgenic model Am J Pathol 2004 165 1289 300 10.1016/S0002-9440(10)63388-3 15466394 PMC1618627

[B12] Breyhan H Wirths O Duan K Marcello A Rettig J Bayer TA APP/PS1KI bigenic mice develop early synaptic deficits and hippocampus atrophy Acta Neuropathol 2009 117 677 85 10.1007/s00401-009-0539-7 19387667

[B13] Lublin AL Gandy S Amyloid-beta oligomers: possible roles as key neurotoxins in Alzheimer's Disease Mt Sinai J Med 2010 77 43 9 10.1002/msj.20160 20101723 PMC3306842

[B14] Tomic JL Pensalfini A Head E Glabe CG Soluble fibrillar oligomer levels are elevated in Alzheimer's disease brain and correlate with cognitive dysfunction Neurobiol Dis 2009 35 352 8 10.1016/j.nbd.2009.05.024 19523517 PMC2725199

[B15] Lefterov I Fitz NF Cronican A Lefterov P Staufenbiel M Koldamova R Memory deficits in APP23/Abca1+/− mice correlate with the level of Abeta oligomers ASN Neuro 2009 1 10.1042/AN20090015 PMC2695585 19570031

[B16] Gouras GK Tampellini D Takahashi RH Capetillo-Zarate E Intraneuronal beta-amyloid accumulation and synapse pathology in Alzheimer's disease Acta Neuropathol 2010 119 523 41 10.1007/s00401-010-0679-9 20354705 PMC3183823

[B17] Dickstein DL Brautigam H Stockton SD Jr Schmeidler J Hof PR Changes in dendritic complexity and spine morphology in transgenic mice expressing human wild-type tau Brain Struct Funct 2010 214 161 79 10.1007/s00429-010-0245-1 20213269 PMC3032082

