# Supplementary material for: The isotropic fractionator provides evidence for differential loss of hippocampal neurons in two mouse models of Alzheimer's disease
Source: Mol Neurodegener. 2012 Nov 22;7:58. doi: 10.1186/1750-1326-7-58 (PMC3551697; doi:10.1186/1750-1326-7-58)
Supplement: Additional file 1 — Table S1. Comparison between the DutchAPPE693Q mouse model and the TgCRND8APPK670N/M671L/V7F mouse model 2345. [file 1750-1326-7-58-S1.docx]

**Supplementary Table 1. Comparison between the DutchAPP^E693Q^ mouse model and the TgCRND8APP^K670N/M671L/V7F^ mouse model**

| Transgenic Line | Mutation | Cognitive deficits | Age at onset of Aβ deposits | Neuronal pathology | Cerebral amyloid angiopathy | Neurofibrillary tangles | References |
| --- | --- | --- | --- | --- | --- | --- | --- |
| Dutch (C57Bl/6J) | APP^E693Q^ | Delayed Acquisition on Morris Water Maze by 12 months of age | Never | Decrease in synaptophysin marker | Present by 12 months of age | Abnormal tau phosphorylation at 8 months | [2-4] |
| TgCRND8  (C3H/He-C57BL/6) | APP^K670N/M671L/V7F^ | Deficits in acquisition and learning reversal on Morris Water Maze by 3 months of age | Present by 3 months of age | 31.8% hippocampal loss by 6 months of age | Present by 15 months of age | Present by 5 months of age | [5]  Present study |
